# Over 3,000 Minimally Invasive Thoracotomies From the European Congenital Heart Surgeons Association for Quality Repairs of the Most Common Congenital Heart Defects: Safe and Routine for Selected Repairs

**DOI:** 10.1177/21501351251322155

**Published:** 2025-03-25

**Authors:** Ali Dodge-Khatami, Juan-Miguel Gil-Jaurena, Jürgen Hörer, Paul Philipp Heinisch, Julie Cleuziou, Sara C. Arrigoni, Robert A. Cesnjevar, Hitendu H. Dave, Alessandro Giamberti, Mauro Lo Rito, Alexander Kadner, Matthias Siepe, Roman Sekelyk, Dmytro Kozhokar, Illya Yemets, Alain Poncelet, Geoffroy de Beco, Jean Rubay, Andre Rüffer, Vincent Kundt, Sameh M. Said, Zdzislaw Tobota, Jeffrey P. Jacobs, Massimo Padalino, Vladimiro Vida

**Affiliations:** 1 Klinik für Kinderherzchirurgie und Chirurgie angeborener Herzfehler, University Hospital RWTH Aachen, Aachen, Germany; 2 Pediatric Cardiac Surgery, Hospital Gregorio Marañón, Madrid, Spain; 3 14924 Pediatric and Congenital Heart Surgery, German Heart Centre Munich, Munich, Germany; 4 Congenital Cardiothoracic Surgery, University Medical Center Groningen, Groningen, the Netherlands; 5 Department of Pediatric Cardiac Surgery, University Children's Hospital, Zurich, Switzerland; 6 Congenital Cardiac Surgery, 27288 IRCCS Policlinico San Donato, Milan, Italy; 7 Inselspital, Universität Bern, Bern, Switzerland; 8 Cardiac surgery, Ukrainian Children’s Heart Center, Kyiv, Ukraine; 9 83415 Institut Cardiovasculaire, Université Catholique de Louvain, Brussels, Belgium; 10 Westchester Medical Center, Maria Fareri Children's Hospital, Valhalla, NY, USA; 11 ECHSA Congenital Database, Warsaw, Poland; 12 Department of Precision and Regenerative Medicine and Jonic Area, University of Bari, Bari, Italy; 13 Department of Cardiac, Thoracic and Vascular Sciences, University of Padova Medical School, Padova, Italy

**Keywords:** Minimally invasive, congenital heart defects, thoracotomy, safety

## Abstract

**Background:**

Minimally invasive thoracotomies to repair selected congenital heart defects are considered only a cosmetic alternative approach by many; however, they represent the routine alternative in centers of expertise.

**Methods:**

Pooled institutional data from the European Congenital Heart Surgeons Association using mini-thoracotomy approaches were analyzed since the beginning of their respective experiences until an inclusion surgical date of January 31, 2024, allowing at least six months postoperative follow-up.

**Results:**

From 1999 to 2024, 3,007 patients from 11 centers underwent surgery. Age and weight ranged from 4 days to 73 years and 3.1 to 106 kg, respectively. Repaired defects included atrial and ventricular septal defects, partial anomalous pulmonary venous return, partial and complete atrioventricular septal defects, double-chambered right ventricle, cor triatriatum, scimitar syndrome, subaortic stenosis, and total anomalous pulmonary venous return (TAPVR). There was no surgical mortality or intraoperative conversion to sternotomy. Complications included wound infections (0.59%), pacemaker requirement (0.23%), phrenic nerve injury (0.26%), bleeding requiring exploration (0.13%), and neurologic injury (0.29%). Early or late reoperations were needed in 0.73%.

**Conclusion:**

Minimally invasive thoracotomies are the preferred approach for selected congenital heart defects in the participating pediatric heart centers. Mini-thoracotomy incisions allow safe access to an ever-expanding spectrum of quality repairs, low morbidity, superior cosmetics, and early return to functionality in infants, children, and adults.

## Introduction

Reducing surgical trauma with increasingly smaller incisions and avoiding a full median sternotomy has long been adopted by our adult cardiac surgery colleagues,^1,2^ whose vast minimally invasive experience has become an established norm and is included in the surgical training curriculum. Through enhanced media exposure, the approach is also gaining momentum within the pediatric sphere, among referring cardiologists, parents, and patients alike. However, there is still reluctance for mini-thoracic approaches to be fully incorporated into the daily armamentarium of pediatric and congenital heart surgeons since it is not an integrated part of the training curriculum. It is often offered only for “simple” defects such as atrial septal defect (ASD) closure, only in certain centers, and only by specific surgeons—but why? As the current generation learns to approach the heart only by using a sternal saw, the trepidation toward any other access is understandable. Yet mini-thoracic approaches are appealing for reasons beyond the obvious cosmetic advantages. The functional components and integrity of the thoracic cage, the cornerstone of which is the sternum, are maintained.^3^ Recognized decades ago by our thoracic surgery colleagues, mini-thoracic incisions are not only cosmetically appealing but lead to enhanced recovery and a quicker return to functionality.^4–6^ As such, mini-thoracic incisions serve a real quality of life purpose which can and *perhaps should be* offered to all patients, if their congenital heart defect anatomy permits it. The current multicenter European Congenital Heart Surgeons Association (ECHSA) experience with over 3,000 patients illustrates the quality of results, and the broadening spectrum of defects and repair types which can be achieved through mini-thoracic incisions.

## Patients and Methods

This retrospective multi-institutional study was submitted to and approved by the ECHSA Research Committee (June 26, 2023, and September 9, 2023, respectively). All participating centers received their local IRB approval to collect and share anonymized retrospective observational data, and parent/patient informed consent was waived.

Patient demographics and perioperative surgical data were analyzed from 3,007 patients undergoing primary repair using cardiopulmonary bypass (CPB) through a mini-thoracotomy incision, operated on between 1999 and a closing enrollment date of January 31, 2024, with a minimum postoperative follow-up time of six months. Defect and repair types were selected by each center/surgeon according to their own experience and comfort levels, with no specific inclusion or exclusion criteria applied across the board. Results are given in mean ± standard deviation and/or median and range when relevant.

## Results

The analysis included 3,007 patients from 11 centers. Median age at surgery was 5.4 years, range 4 days to 73 years. Median weight was 18.9 kg, range 3.1 to 106 kg. Female patients represented 56.5% (n = 1700/3007) of the patient population, prematurity was present in 4.7% (n = 102/2,166 available), and chromosomal anomalies in 4.5% (n = 97/2,172 reported). Primary diagnoses and repair types are given in Tables 1 and 2. While the case mix reflected a predominant percentage of patients with ASD (n = 2211/3007, 73.5%) and partial anomalous pulmonary venous return (PAPVR) (n = 466/3,007,15.5%), repairs across the entire board (mostly influenced by case numbers in seven centers), ventricular septal defect (VSD) closures, repair of tetralogy of Fallot along with both partial and complete AVSD repairs represented nearly a third of all repairs (n = 140/435; 32.2%) in 4 “avant-garde” centers using more aggressive selection criteria, stemming from longer experience, and/or comfort levels with the approach (Table 3). Excluding blood used for circuit priming, perioperative transfusion of blood products at any time after coming off bypass (ie, intraoperatively by anesthesia, on the intensive care unit, or prior to hospital discharge), was required in 6.4% of patients (n = 185/2,889 documented). Unplanned reoperations for residuals, either during the same hospital stay or during follow-up, were needed in 22/3,007 patients (0.73%), at a mean interval of 154.6 ± 410.9 days, ranging from the day of operation to four years postoperatively. These included larger residual left-to-right shunts in sinus venosus ASD (n = 4) or ventricular septal defect (n = 1) requiring patch revision, ASD patch dehiscence or interatrial septal tear after direct closure technique (without patch) requiring revisions (n = 9) or occluder-device implantation (n = 2), residual mitral insufficiency requiring revision (n = 4), pleurodesis/thoracic duct ligation for chylothorax (n = 1), and creation of an ASD fenestration after tetralogy of Fallot repair (n = 1). Early reoperations (during the initial hospital stay of index surgery; n = 13) were all performed through the same mini-thoracotomy incision, along with one early catheter-based device insertion. Of the five late reoperations specified below, three were performed through the prior thoracotomy incision, and two required sternotomy; three patients underwent catheter-based late reinterventions.

**Table 1.** Primary Diagnoses Undergoing Repair (n = 3,007).

	n (%)
ASD	2,211 (73.5)
Sinus venosus ASD + partial anomalous pulmonary venous return	466 (15.5)
VSD	139 (4.6)
Partial atrioventricular canal (primum ASD + left AV valve cleft)	109 (3.6)
Complete atrioventricular septal defect	9 (0.3)
Mitral valve insufficiency	19 (0.6)
Aortic stenosis/insufficiency—subaortic stenosis	17 (0.6)
Cor triatriatum	11 (0.4)
Double-chambered right ventricle	7 (0.2)
Tetralogy of Fallot	7 (0.2)
Hypoplastic right ventricle/Ebstein	3 (0.1)
Total anomalous pulmonary venous return	3 (0.1)
Scimitar syndrome	2 (0.1)
Coronary fistula	1
Tricuspid valve insufficiency	3 (0.1)

Abbreviations: ASD, atrial septal defect; AV, atrioventricular; VSD, ventricular septal defect.

**Table 2.** Primary Procedures Performed (n = 3,007).

	n
Patch closure for ASD, VSD, pAVC, or cAVSD	1,965
Direct (primary) closure for ASD, VSD	762
Double patch technique for PAPVR or cAVSD	187
Warden operation for PAPVR	31
Mitral or tricuspid valve repair	15
Mitral/tricuspid replacement, RV-PA conduit	7
Subaortic stenosis/DCRV (membrane/muscle resection)	20
Bidirectional Glenn	3
TAPVR repair	3
Tetralogy of Fallot	5
Left-sided PAPVR-LAA anastomosis	8
Coronary fistula repair	1

Abbreviations: ASD, atrial septal defect; cAVSD, complete atrioventricular septal defect; DCRV, double-chambered right ventricle; LAA, left atrial appendage; PA, pulmonary artery; PAPVR, partial anomalous pulmonary venous return; pAVC, partial atrioventricular canal = primum ASD + left atrioventricular valve cleft; RV, right ventricle; TAPVR, total anomalous pulmonary venous return; VSD, ventricular septal defect.

**Table 3.** Primary Diagnoses Undergoing Repair in 4 “Avant-Garde” Centers (n = 435): Median Age 4.2 Years (4 Days to 66.5 Years), Median Weight 15.9 kg (3.1-106).^a^

	n = 435 (%)
ASD	218 (50.1)
Sinus venosus ASD + partial anomalous pulmonary venous return	54 (12.4)
**VSD**	**100** (**23)**
**Partial atrioventricular canal (primum ASD + left AV valve cleft)**	**27** (**6.2)**
**Complete atrioventricular septal defect**	**7** (**1.6)**
Mitral valve insufficiency	3 (0.7)
Aortic stenosis/insufficiency—subaortic stenosis	4 (0.9)
Cor triatriatum	6 (1.4)
Double-chambered right ventricle	4 (0.9)
**Tetralogy of Fallot**	**6** (**1.4)**
Hypoplastic right ventricle/Ebstein	1 (0.2)
Total anomalous pulmonary venous return	2 (0.5)
Scimitar syndrome	1 (0.2)
Coronary fistula	
Tricuspid valve insufficiency	2 (0.5)

Abbreviations: ASD, atrial septal defect; AV, atrioventricular; VSD, ventricular septal defect.

^a^ Central image: Minimal Invasive Surgery Working Group of the European Congenital Heart Surgeons Association (ECHSA) and participating centers for the study.

Of the total population of 3,007 patients, postoperative complications included phrenic nerve injury in 8 patients (0.26%), advanced degree heart block requiring a pacemaker in 7 (0.23%), new-onset arrhythmia requiring medication beyond hospital stay in 10 (0.33%), bleeding requiring surgical reexploration in 4 (0.13%), wound infection or dehiscence including either the thoracotomy or groin cannulation site in 18 (0.59%), and a new persisting neurological deficit/stroke in 9 (0.29%).

Incisions included the horizontal axillary in 1,866 (62.1%) patients, a vertical midaxillary in 884 (29.4%), a submammary in 241 (8%), and a posterolateral in 16 (0.5%).

Cannulation for CPB was central (aortobicaval) in 2,294 patients (76.3%), peripheral vascular (femoral/iliac artery and vein) in 396 (13.2%), and combined central/peripheral in 317 (10.5%). Complications due to peripheral vascular cannulation occurred in 23/713 patients (3.2%), ranging from groin hematoma, seroma or wound infection, or mild/moderate femoral vascular stenosis.

Induced ventricular fibrillation (VF) was used in 2,001 patients (66.5%) and aortic cross-clamping with cardioplegic arrest in 998 (33.2%). The eight remaining patients required neither (left-sided PAPVR without an associated intracardiac shunt performed with bypass on a beating heart). Median induced ventricular fibrillation, aortic cross-clamp, and CPB times were 18 min (range 3-120), 35 min (range 5-214), and 45 (range 3-400), respectively. There were no conversions to sternotomy; one horizontal axillary incision was prolonged posteriorly to form a posterolateral incision (1/3,007, 0.03%).

All patients (n = 3007) received standard postoperative pain management including intravenous opioids with oral analgesia: in isolation for 2,313 patients (76.9%), in combination with an intercostal block/catheter in 494 (16.4%), with an epidural catheter in 140 (4.7%), with a regional serratus block in 56 (1.9%), or with an erector spinae/caudal block in 4 cases (0.1%).

The median length of intubation time was 4 h (range 1-216) in 1,442 patients (48% of all patients). In-theater extubation protocols leading to “zero” intubation times were achieved in 1,565 patients (52%). The median length of chest tube drainage was one day (range 1-72), of intensive care stay one day (range 1-30), and of hospital stay eight days (range 1-172).

There was no surgical mortality; one late death occurred six years postoperatively at the age of 79. A minimal follow-up time of six months postoperatively was requested to exclude/capture major events or morbidity potentially related to surgery, available in all 3,007 patients. At a median follow-up time of 2.1 years (range 6 months-21.8 years), 1,556/1,640 patients (94.9%) were in NYHA/Ross class I, 83/1,640 (5.1%) in class II, and one patient was in class III. No information beyond six months was available in 1,367 patients (45.5%). There were eight recorded late reoperations/reinterventions (beyond the initial index operation hospital stay): one transvenous pacemaker insertion for heart block (after three years), one ASD patch dehiscence after partial AVC repair requiring revision through the same right axillary thoracotomy incision (after ten months), three residual ASDs after ASD repair revised through the same thoracotomy incision (after 28 days), closure by an occluder device in one patient (after 6.4 months) and reoperated through sternotomy in another (after four years), one stent of the superior vena cava (SVC)-right atrial junction after a double-patch PAPVR repair (after three years), and two redo mitral valve repairs for insufficiency following repairs of partial AVC (after two years through median sternotomy) and complete AVSD (after two months through the initial right axillary incision).

**Figure 1.**
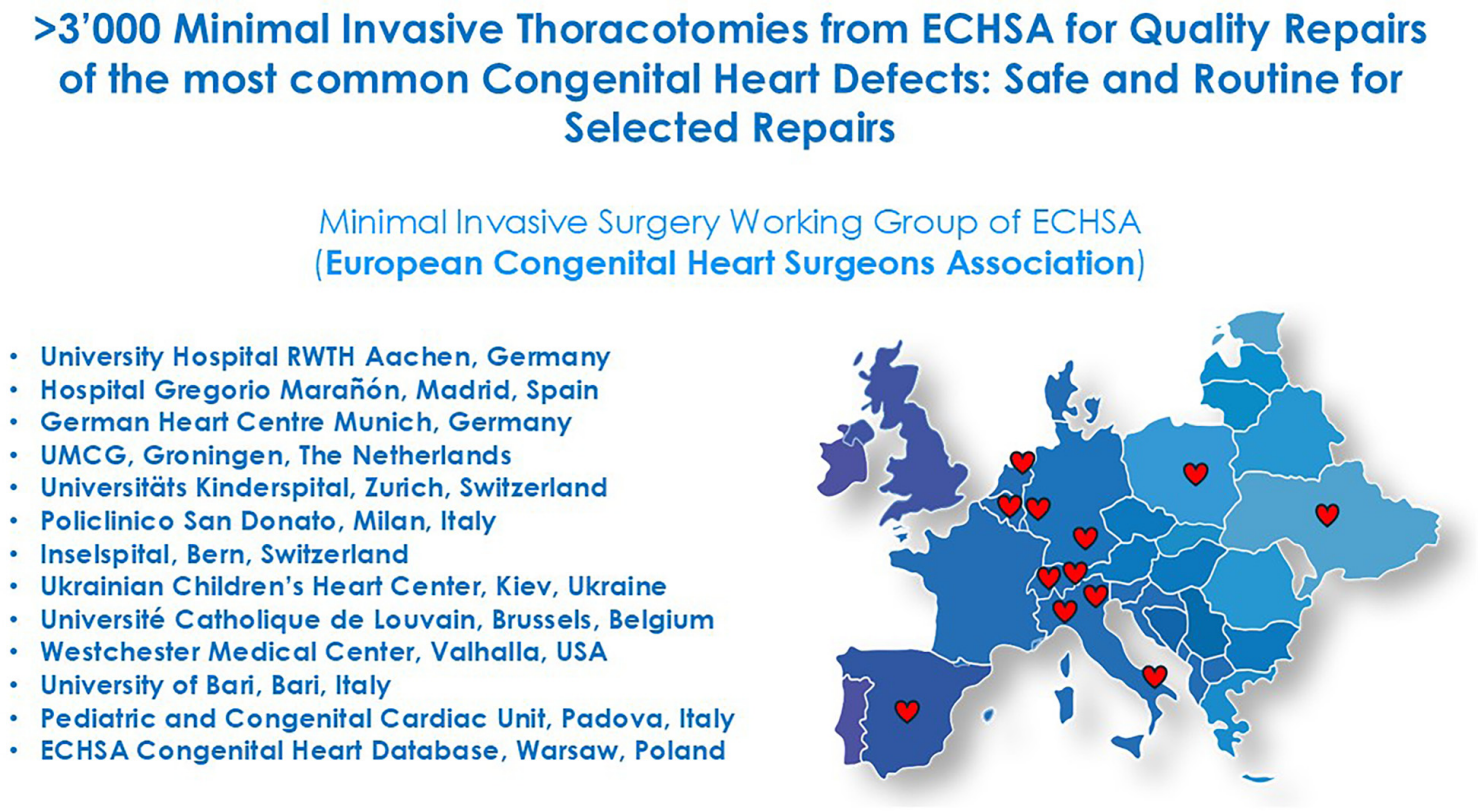
Central Image: Minimally Invasive Surgery Working Group of the European Congenital Heart Surgeons Association (ECHSA) and participating centers for the study.

## Discussion

Historically, although the first closed and then open-heart repairs using CPB were performed through a median sternotomy, virtually all cardiac surgeons of the early 1950s preferred the bilateral anterior thoracotomy or clamshell incision, from fear of lack of exposure, and for safe caval cannulation.^7^ First described by Milton for anterior mediastinal and tracheal access in 1897,^8^ reintroduced by Shumacker^9^ in 1953 for closed cardiac procedures, and finally popularized by Julian for open heart surgery on CPB in 1957,^10^ the median sternotomy has gained widespread recognition, and universally taught as the standard approach to performing cardiac surgery. As a result, the sternotomy saw is somehow currently perceived as a first-line instrumental prerequisite to perform open heart surgery.

However, operations performed through mini-thoracotomies, be it right or left, are not new, and in fact the first report of an open-heart operation (VSD closure) using aortic occlusion and potassium citrate arrest during CPB was performed in 1956 through a transverse anterior right thoracotomy, by Effler et al.^11^ It is therefore ironic and regrettable that the approach is no longer routinely taught, making thoracotomy approaches a domain reserved for a marginal few, and thought to be at higher risk, given the lack of space and easy access to all cardiac structures, compared with the comfort zone offered through median sternotomy. The current ECHSA study with more than 3,000 operations performed by 22 different surgeons in various settings reports on quality repairs with no mortality, no need for intraoperative conversion to sternotomy, and a very low incidence of unplanned reoperations for residuals or complications. In itself, this is a testimony to the safety of the approach, which should debunk any commentary to the contrary. The mini-thoracotomy is being performed for an ever-expanding number of congenital heart defect diagnoses, not only for “simple” ASD closures but also for RACHS-1 scores 2 (bidirectional Glenn), 3 (Ebstein repair, left outflow tract procedures, partial and complete atrioventricular septal defect repair), and 4 categories (modified Konno procedure, TAPVR repair). For more more than two decades, this has been the result of tremendous courage, grit, tenacity, discipline, and patience of many surgeons, who are paving the way for the extant generation to follow in safe footsteps.

### Incisions

Various incisions are used to approach the right and left cardiac structures, depending on the type of repair and defect. Typically, through a right thoracotomy, ASDs, right-sided PAPVR, VSDs, double-chambered right ventricle, partial or complete AVSD, scimitar syndrome, cor triatriatum, bidirectional Glenn, tricuspid (Ebstein or not) and mitral valve repairs, subaortic stenosis or aortic valve repair, milder variants of tetralogy of Fallot, or more recently cardiac TAPVR, can be repaired.^12–19^ From the left chest, left-sided PAPVR, tetralogy of Fallot, pulmonary valve replacement, and RV-PA conduit exchange have been performed.^15,20^

The rate of postoperative surgical site wound infections in this series, including both the thoracic incisions and the groin cannulation sites, was 0.6%. This compares very favorably to that experienced after repairs through median sternotomy, ranging between 0.25% and 6% in a recent North American study.^21^ Early mobilization is encouraged, and no limitations to physical activity are imposed on infants, children, or adult patients after hospital discharge, which is a relief for the patients and parents alike.^22–24^

Although encountered in only a minority of our series (4.5%, n = 97/2,172 reported), this approach is offered to infants and children with common (Down) and less common syndromes (Holt-Oram, Trisomy 18), as avoiding a sternotomy in these patient populations may offer functional advantages (no sternal dehiscence or deformities from postoperative hyperactivity leading to sternal syndesmosis, pectus excavatum,^25^ or carinatum^25,26^), and not just a cosmetic one.^25^ With increased technical and physical comfort using tips and tricks to enhance exposure in the surgical field,^27^ the weight and age have dramatically decreased, with repairs being performed in babies as young as four days and weighing as little as 3.1 kg.

For mini incisions, induced ventricular fibrillation may allow more expeditious repairs with the specific advantage of avoiding cumbersome instruments such as cross-clamps and cardioplegia needles, which take up precious space in the surgical field.^27^ This may be done using either a fibrillation probe which is sutured to the right ventricular epicardium and connected to a fibrillatory device or by using temporary epicardial pacing wires secured to the right ventricular wall and attached to an atrial overdrive (>300 bpm) pacing device. This was performed in 8/11 centers in 66.5% of all cases (n = 2,001/3007), with a mean time of 18 ± 10 min (range 3-120). Although induced ventricular fibrillation has been around for a very long time prior to the advent of cardioplegia,^28^ it has unfortunately disappeared from the regular classic training curriculum. Therefore, many younger surgeons are either unaware or uncomfortable with it, feeling unable to operate in the absence of an aortic cross-clamp. There is indeed a learning curve required for induced VF and the additional de-airing maneuvers prior to defibrillating, which are however outweighed by its advantages once a comfort level has been reached.^29^ It is critical to surgically secure continuous contact between the probe or wire and the heart prior to de-airing maneuvers, as unwanted premature spontaneous defibrillation from loss of contact may potentially lead to devastating air embolism and complications. While the “safe” interval to use induced ventricular fibrillation at moderate hypothermia is not defined and could be pushed to around an hour,^30^ most surgeons will probably select a more reliable method for myocardial protection, such as cross-clamping and cardioplegia, when the anticipated time for repair extends beyond 40 min.^31^

### Cannulation, Pitfalls, Trends, and Low Complication Rates

Central cannulation through mini-thoracotomies was performed in 76.3% (2297/3007) of cases. In 23.7% (715/3007) of cases some form of peripheral cannulation; that is, femoral or iliac arteries or veins, or more rarely percutaneous internal jugular vein cannulation was used. Complications due to peripheral cannulation, including not only vascular stenosis or thrombosis but also groin hematoma or seromas, occurred in 3.2% (23/713) of our cohort. Our rate is somewhat higher than that of Wadhawa et al,^32^ who reported on 200 children at a median age of six years (range 3-18) weighing 15 kg (range 8-45), in whom systematic femoral artery and vein were cannulated, along with the SVC. There were no major complications related to peripheral cannulation, and no significant femoral artery or vein stenosis at discharge or one-year follow-up on color Doppler.^32^ Complications due to cannulation of peripheral vessels in patients of any size are certainly to be avoided at all costs and should not become an Achille's heel of the minimally invasive approach. It is incumbent upon us to either strive for systematic central cannulation or to apply meticulous surgical technique with or without chimney grafts to peripheral arteries, in order not to tarnish the reputation of the approach, by trading enhanced cosmetics for peripheral vascular consequences.

### Pain Control, Myths, and Reality

Unfortunately, thoracotomies are perceived by many to be more painful than the median sternotomy. This is largely based on outdated literature associated with either clamshell incisions (bilateral thoracotomies that also involve splitting part of the sternum)^6^ or old-school, nonmuscle-sparing, and aggressive rib fracturing using large incisions.^7^ In the modern era, newer surgical techniques and school of thought strive for shorter incisions, muscle-sparing approaches, gentle rib spreading without fracturing the ribs, meticulous care for reapproximation of the rib spaces, and chest wall closure, all of which are aimed at less invasiveness, and hence at least an attempt at prophylaxis toward less pain.^13,27^ Adjuncts at reducing the quantity of oral and intravenous opioids and anti-inflammatory agents include various types of local anesthesia regional blocks after induction of anesthesia.^33^ These include intercostal, epidural, ultrasound-guided serratus anterior, and erector spinae blocks,^33,34^ mostly using 3 mg/kg of 0.2% ropivacaine. As an adjunct to standard intravenous and oral pain medication, local anesthetic blocks were used in 23.1% of our cases.

### Study Limitations

The descriptive observational nature of the study is an inherent limitation of a retrospective analysis. There is neither control group nor generalized inclusion or exclusion criteria to determine suitability for the minimally invasive approach, compared with treatment for the same defects through median sternotomy or by percutaneous device closure. This takes away the denominator for each defect type in each center and throughout the collective, which was not addressed as an epidemiologic question to the centers. The study was not designed for long-term follow-up but was meant to capture any events related to surgery up to at least six months postoperatively, available in all patients. Beyond six months, there was follow-up in 54.5% of the entire cohort.

In conclusion, since 1999, the extant ECHSA study of 3,007 successful repairs of the most common congenital heart defects through mini-thoracic incisions throughout 11 centers by 22 surgeons represents, to our knowledge, the largest report to date of an alternative approach to median sternotomy. With no mortality or conversion to another incision, an extremely low unplanned or delayed reoperation rate, and very low complications, it may reasonably be stated that the results using the mini-thoracic incisions are excellent, the approach is safe, and are the routine alternative for selected defects and repairs in the participating centers.

## Abbreviations

ASD: atrial septal defect
CPB: cardiopulmonary bypass
ECHSA: European Congenital Heart Surgeons Association
PAPVR: partial anomalous pulmonary venous return
SVC: superior vena cava
VSD: ventricular septal defect
